# DDX19A Promotes Metastasis of Cervical Squamous Cell Carcinoma by Inducing NOX1-Mediated ROS Production

**DOI:** 10.3389/fonc.2021.629974

**Published:** 2021-04-22

**Authors:** Yanhui Jiang, Baibin Wang, Yongliang Li, Jiahui Shen, Yutao Wei, Hanjie Li, Shangqiu Chen, Hua Yang, Famin Zeng, Changqing Liu, Feng Wang, Huanhuan He, Yong Chen, Jihong Liu

**Affiliations:** ^1^Department of Gynecology, The Fifth Affiliated Hospital of Sun Yat-sen University, Zhuhai, China; ^2^Guangdong Provincial Key Laboratory of Biomedical Imaging, Guangdong Provincial Engineering Research Center of Molecular Imaging, The Fifth Affiliated Hospital of Sun Yat-sen University, Zhuhai, China; ^3^Department of Pathology, The Fifth Affiliated Hospital of Sun Yat-sen University, Zhuhai, China; ^4^Department of Interventional Medicine, The Fifth Affiliated Hospital of Sun Yat-sen University, Zhuhai, China; ^5^Department of Gynecologic Oncology, State Key Laboratory of Oncology in South China, Sun Yat-sen University Cancer Center, Guangzhou, China

**Keywords:** DDX19A, cervical squamous cell carcinoma, metastasis, Nox1, reactive oxygen species

## Abstract

The major obstacle to treat cervical squamous cell carcinoma (CSCC) is the high prevalence of metastasis, which severely affects 5-year survival rate and quality of life for cancer patients. The DEAD-box helicase family has been reported to be a critical mediator in the development and metastasis of various cancers. DEAD-box helicase 19A (DDX19A) is a member of the DEAD-box helicase family; however, its functional role in CSCC is unclear. In this study, bioinformatics analysis of clinical samples from public databases demonstrated that the expression of DDX19A was elevated in CSCC tissues and that high expression of DDX19A was positively correlated with metastasis and poor clinical outcome. Functionally, we found that DDX19A promoted CSCC cell migration and invasion *in vitro* and lung metastasis *in vivo*. Mechanistically, overexpression of DDX19A increased NADPH oxidase 1 (NOX1) expression, enhanced reactive oxygen species (ROS) production, and induced the migration and invasion of CSCC cells. Rescue experiments revealed that DDX19A-induced CSCC functional alterations were dependent on NOX1 and that DDX19A-promoted CSCC metastasis was abrogated upon the inhibition of ROS. Our results demonstrated that DDX19A could promote CSCC metastasis by inducing NOX1-mediated ROS production and that blockage of the NOX1/ROS axis might serve as a potential therapeutic target for patients with DDX19A-overexpressed CSCC.

## Introduction

Cervical cancer is one of the most common gynecological malignancies and the second leading cause of cancer-related deaths among women worldwide (1). Thanks to advances in surgery, chemotherapy, radiotherapy, and immunotherapy, the 5-year survival rate for patients with cervical cancer has increased to 80%. However, once local metastasis or distant metastasis occurs, the 5-year survival rate slumps to ~50% (2). Metastasis is the primary cause of death in patients with cervical cancer (3, 4), and cervical squamous cell carcinoma (CSCC) is the most common pathologic type of cervical cancer, accounting for ~80% of all cases (5). Thus, it is imperative to understand the underlying mechanism regarding the metastasis of CSCC and to identify novel targets and therapies.

The DEAD-box family, characterized by the conserved Asp-Glu-Ala-Asp (DEAD) motif, represents a large group of RNA helicases consisting of 37 members (6, 7). DEAD-box proteins are involved in various RNA metabolic processes including transcription, RNA transport, and RNA degradation (8–10). Recent studies have revealed that many DEAD-box proteins were abnormally expressed and play pivotal roles in cancer metastasis (11–14). For example, DEAD-box helicase 39 (DDX39) could promote hepatocellular carcinoma progression and metastasis by activating the Wnt/β-catenin pathway (15), and DDX3 activated CBC-eIF3-mediated translation of uORF-containing oncogenic mRNAs to promote metastasis in head and neck squamous cell carcinoma (16). On the contrary, DDX1 could inhibit ovarian tumor metastasis through regulating primary microRNA maturation (17). However, the expression and function of DEAD-box proteins in CSCC remain unknown.

DDX19A, a member of the DEAD-box family, was identified as a novel cytosolic RNA sensor that could activate the NLRP3 inflammasome during virus infection (18). DDX19A was proven to be associated with NADPH oxidase 1 (NOX1)-mediated oxidative stress in tumor necrosis factor (TNF)-α-induced A549 cells (19). The expression and function of DDX19A in tumor development have not been defined. Interestingly, immunohistochemical (IHC) staining data from the Human Protein Atlas website (https://www.proteinatlas.org/) indicate that the expression of DDX19A protein was highest in cervical cancer among all the common cancer types. According to data from The Cancer Genome Atlas (TCGA) and Gene Expression Omnibus (GEO) websites, DDX19A was found highly expressed in CSCC samples compared with normal cervical tissues. High expression of DDX19A was positively correlated with metastasis and poor clinical outcome of patients. Therefore, we attempt to verify the function and investigate the underlying mechanism of DDX19A in the metastasis of CSCC. Our findings may provide a novel therapeutic target of CSCC.

## Materials and Methods

### Clinical Tissues

A tissue microarray (TMA) containing 76 pairs of CSCC tissues, non-adjacent normal tissues, and 10 non-paired CSCC tissues was constructed with clinical specimens obtained between January 2008 and February 2016 from the Fifth Affiliated Hospital of Sun Yat-sen University. The diagnosis of CSCC was confirmed by pathologists, and the study was approved by the Institutional Research Ethics Board of the Fifth Affiliated Hospital of Sun Yat-sen University. Patients had not received any chemotherapy or interventional treatments prior to surgical resection. General information and clinical characteristics of the CSCC patients are provided in Supplementary Table 1.

### Cell Culture

The human CSCC cell lines, HCC94, CaSki, C33A, SiHa, and MS751, were purchased from the American Type Culture Collection (ATCC; Manassas, VA, USA). HCC94, CaSki, C33A, and SiHa cells were grown in Dulbecco's modified Eagle's medium (DMEM) supplemented with 10% fetal bovine serum (FBS; Life Technologies). MS751 cells were grown in RPMI-1640 medium (Life Technologies, Carlsbad, CA, US) supplemented with penicillin G (100 U/ml), streptomycin (100 mg/ml), and 10% FBS (Life Technologies). All cell lines were cultured at 37°C in a humidified atmosphere of 5% CO_2_.

### Quantitative Real-Time PCR

Total RNA was isolated using TRIzol followed by DNase (Invitrogen) treatment. qRT-PCR was performed using SYBR Green master mix (Vazyme Biotech Co., Ltd., Nan Jing, China) on a Bio-Rad iCycler. The primers used for qRT-PCR were listed as follows: DDX19A forward primer: 5′-CATGGGCTTCAATCGACCCT-3′, reverse primer: 5′-GCACAGACACTGGGGGTATC-3′; NOX1 forward primer: 5′-GTTTTACCGCTCCCAGCAGAA-3′, reverse primer: 5′-GGATGCCATTCCAGGAGAGAG-3′; GAPDH forward primer: 5′-AGGGCTGCTTTTAACTCTGGT-3′, reverse primer: 5′-CCCCACTTGATTTTGGAGGGA-3′.

### Protein Isolation and Western Blot Analysis

Protein samples were prepared from cell lysates, and the protein concentration was determined using a bicinchoninic acid (BCA) kit (Beyotime Biotechnology, Beijing, China). The proteins were separated by 10% sodium dodecyl sulfate-polyacrylamide gel electrophoresis (SDS-PAGE) and transferred to nitrocellulose membrane. The membrane was blocked with 5% nonfat milk in phosphate-buffered saline (PBS) for 1 h at room temperature and then incubated overnight at 4°C with primary antibody: anti-DDX19A (#ab235531, 1:1,000, Abcam), anti-NOX1 (#17772-1-AP, 1:1,000, Proteintech), and anti-GAPDH (#60004-1, 1:1,000, Proteintech). The membranes were washed and incubated with the corresponding horseradish peroxidase (HRP) secondary antibodies for 1 h at room temperature. Finally, the protein signals were detected semiquantitatively using the SuperSignal™ West Pico PLUS Chemiluminescent Substrate (Thermo Scientific™).

### Immunohistochemical Staining

Seventy-six pairs of CSCC and adjacent normal tissues and 10 non-paired CSCC tissues were used to prepare a TMA. After incubating at 60°C for 2 h, the tissues were deparaffinized with dimethylbenzene and rehydrated in different concentrations of alcohol. To retrieve antigen, the slides were heated at 95°C in 0.01 M citrate buffer (pH 6.0), and 3% hydrogen peroxide was used to quench peroxidase activity for 20 min. To avoid non-specific staining, the tissues were incubated with normal goat serum, followed by incubation overnight with anti-human DDX19A (HPA045252, 1:250, Sigma) or anti-human NOX1 (#GTX103888-S, 1:100, GeneTex) at 4°C. After rinsing with PBS, the tissues were incubated with a secondary antibody for 1 h and stained with 3,3′-diaminobenzidine (DAB; Zhongshan Biotech, Beijing, China). After hematoxylin counterstaining, the sections were dehydrated and sealed. Two experienced pathologists independently evaluated the percentage of positive tumor cells and the staining intensity. The values for DDX19A and NOX1 staining intensity were assigned as follows: 0 (negative), 1 (weak), 2 (moderate), and 3 (strong). The values for the percentage of positive tumor cells were scored as follows: (1) (0–25%), (2) (26–50%), (3) (51–75%), and (4) (76–100%). The immunoreactive score (IRS) for each section was calculated by the product of the staining intensity and the percentage of tumor cells.

### Cell Migration and Invasion Assays

Cell migration and invasion assays were performed as previously described (20). Transwell chambers (Corning, Corning, NY, USA) equipped with 8-μm pore insets were used for the migration and invasion assays. For the migration assay, Transwell chambers (Corning, Corning, NY, USA) containing 8-μm pores were uncoated with Matrigel in the upper chamber. For the invasion assay, Transwell chambers (Corning, Corning, NY, USA) containing 8-μm pores were coated with 100 μl of 1:8-diluted Matrigel (BD Biosciences) in the upper chamber. Briefly, 100 μl of cell suspension (8 × 10^4^ cells) of serum-free medium was plated in the upper chamber; medium with 10% FBS was added to the lower chamber. After incubating 18 h for migration and 22 h for invasion, we removed the cells of the upper chamber and then stained the migration and invasive cells of the lower chamber using crystal violet solution. The results were photographed by light microscopy and counted by ImageJ. The experiment was done in triplicate.

### Measurement of Cellular Reactive Oxygen Species

ROS levels in the human CSCC cell lines were measured using the Reactive Oxygen Species (ROS) Assay Kit (Beyotime, China) following the manufacturer's protocol. CSCC cells were incubated with 2′,7′-dichlorofluorescin diacetate (DCFH-DA) probe at 37°C for 20 min and washed three times with serum-free DMEM medium. DCFH-DA fluorescence was measured using a Micro Fluorescence Reader with an excitation wavelength of 488 nm and an emission wavelength of 525 nm.

### Plasmids, Retroviral Infection, and Transfection

DDX19A overexpression and knockdown by shRNA were conducted by a lentiviral infection system, which was purchased from Shanghai Genechem Co., Ltd. (Shanghai, China). Briefly, lentiviral vector and 1 μg/ml polybrene were added to infect CSCC cells in growth medium for 12–24 h. Then, the growth medium of the lentiviral vector-infected CSCC cells was removed, and fresh growth medium was added to the culture dish for 24–48 h. Finally, 2 μg/ml puromycin was added to the growth medium of CSCC cells for selecting lentiviral vector-infected cells to obtain stable expression clones. Plasmid transfection and siRNA transfection were performed using Lipofectamine LTX reagent (Invitrogen, Carlsbad, CA) according to the manufacturer's instruction. Briefly, 1 μg DNA and 5 μl of transfection reagent were mixed in serum-free growth medium and added into a 60–90% confluent cell layer. Plasmid, siRNA, and shRNA were used in this study as follows: (a) DDX19A lentiviral vector U6-sh-DDX19A-EGFP-IRES-puromycin; (b) DDX19A-specific short hairpin RNA (RNA#1: 5′-GTACTCGGTGAAGTCGTTTT-3′ and RNA#2: 5′-CTGTCAAGTCGATGACCAA-3′); (c) DDX19A-specific siRNA (siRNA#1: 5′-GATCGTGACTCCCACTGTA-3′ and siRNA#2: 5′-GGCAGTATATCTTTGTTAA-3′); (d) NOX1-specific siRNA (siRNA#1: 5′-CTGTCAAGTCGATGACCAA′ and siRNA#2: 5′-TGTCAAGTCGATGACCAAT′); (e) DDX19A or NOX1 overexpression vector pcDNA3.1-DDX19A (pcDNA3.1-NOX1) and control vector plasmids were designed and synthesized by RiboBio (Guangzhou, China).

### *In vivo* Metastasis Experiment

BALB/c nude mice (4–5 weeks of age, female, 20–24 g) were purchased from Vital River Lab Animal Technology Co., Ltd., housed under standard conditions at the animal care facility at Guangdong Provincial Key Laboratory of Biomedical Imaging. BALB/c nude mice were used for tail vein injection experiments to evaluate the metastatic ability of CSCC cells *in vivo*. SiHa cells with DDX19A stable knockdown (1 × 10^6^) or negative control SiHa cells (1 × 10^6^), and SiHa cells overexpressing DDX19A or control SiHa cells, were injected into the tail veins of nude mice. After 10 weeks of caudal intravenous injection, the lungs were harvested and weighed, and the number of metastatic nodules was counted on the surface of the lungs. Then, lungs were fixed in 4% formalin and embedded in paraffin, and 5-μm sections were stained with H&E. Animal protocols were approved by the Institutional Animal Care and Use Committee of the Fifth Affiliated Hospital of Sun Yat-sen University.

### Bioinformatics and Data Analyses

DDX19A mRNA expression in normal cervical tissues and CSCC tissues were examined according to TCGA (https://gdc.cancer.gov/) and the GEO dataset (GSE7803) (see URL https://www.ncbi.nlm.nih.gov/geo). Gene expression was presented as the mean value of multiple probes for each gene after log2 transformation. Comparisons (normal cervical tissues vs. CSCC tissues) were analyzed by the Mann–Whitney *U*-test. *p* < 0.05 was considered statistically significant.

### Statistical Analysis

Results are presented as the mean ± SEM. All statistical analyses were performed using GraphPad Prism 5 software (GraphPad software, Inc., La Jolla, CA). Data from three independent experiments were expressed as the mean ± SD. A value of *p* < 0.05 was considered to be significant, *p* < 0.05, *p* < 0.01, and *p* < 0.001 are designated as ^*^, ^**^, and ^***^, respectively.

## Results

### DDX19A Is Upregulated in Cervical Squamous Cell Carcinoma Tissues and Is Associated With Poor Prognosis in Cervical Squamous Cell Carcinoma Patients

**Figure 1:**
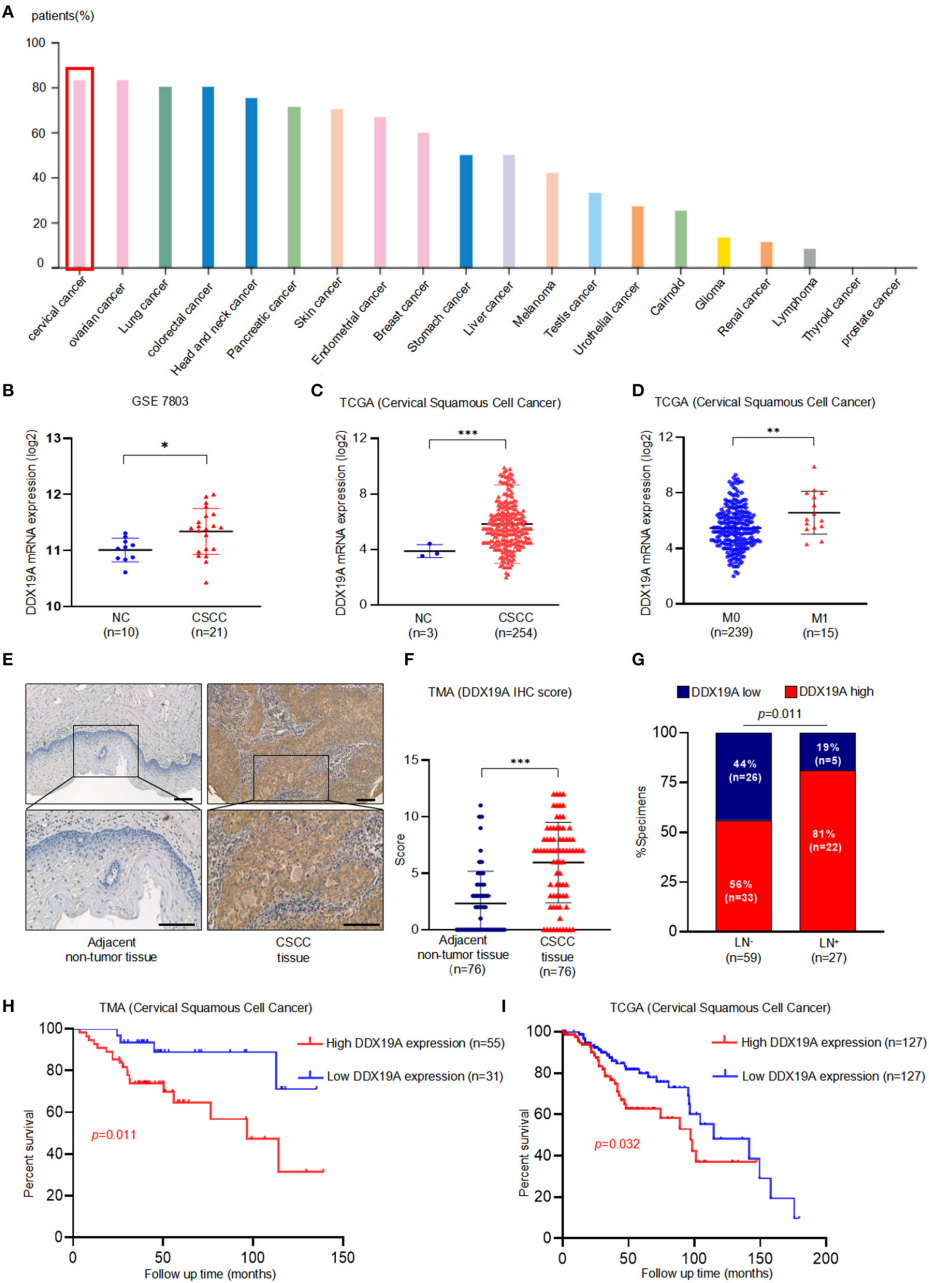
DEAD-box helicase 19A (DDX19A) is upregulated in cervical squamous cell carcinoma (CSCC) tissues and is associated with poor prognosis in CSCC patients. **(A)** DDX19A protein expression in 20 types of human cancers on Human Protein Atlas website (https://www.proteinatlas.org). **(B,C)** The expression level of DDX19A mRNA in GSE7803 database and The Cancer Genome Atlas (TCGA) database (NC, normal cervix; CSCC, cervical squamous cell carcinoma). **(D)** The expression level of DDX19A mRNA between metastasis (M1) and non-metastasis (M0) in TCGA database (CSCC). **(E)** Representative images of the immunohistochemical (IHC) staining of DDX19A in human CSCC tissue and adjacent non-tumor tissues (200× and 400× magnification; scale bar: 200 μm). **(F)** Dot plots to show the IHC score of DDX19A expression using 76 pairs of CSCC tissues and adjacent non-tumor tissues tissue microarray (TMA) tissue sections (*p* < 0.001). **(G)** Correlation between lymph node metastasis and DDX19A expression in CSCC patients. Chi-square test was used. **(H)** Kaplan–Meier analysis was performed for our CSCC patients' cohort to evaluate the association between DDX19A protein expression and 86 CSCC patients' overall survival. **(I)** Kaplan–Meier analysis was performed for CSCC patients' cohort in TCGA database (CSCC) to evaluate the association between DDX19A mRNA level and patients' overall survival. Results were shown as means ± SD, *p < 0.05, **p < 0.01, ***p < 0.001 by Student's *t*-test. NS, not significant.

IHC staining data from the Human Protein Atlas website (https://www.proteinatlas.org/) indicated that the expression of DDX19A protein was highest in cervical cancer among all the common cancer types (Figure 1A), suggesting a pivotal role for DDX19A in this gynecological malignancy. Furthermore, microarray analysis [GEO dataset (GSE7803) and TCGA (CSCC data)] revealed that the expression of DDX19A mRNA was upregulated in CSCC samples compared with that in normal cervical tissues (Figures 1B,C). In-depth analysis of TCGA database showed that DDX19A expression was higher in CSCC with distant metastasis (M1) than that in specimens without metastasis (M0) (Figure 1D).

In order to validate these findings, the protein expression of DDX19A was examined in 76 pairs of CSCC and adjacent normal tissues and 10 non-paired CSCC tissues by TMA. Our results showed that CSCC tissues had significantly higher staining intensity of DDX19A compared with the adjacent non-tumor tissues (Figures 1E,F, Supplementary Table 2). To further assess the clinical significance of DDX19A expression, we divided 86 CSCC cases into low-expression (*n* = 31) group and high-expression (*n* = 55) group, with the cutoff value defined as the median of the IHC immunoreactive score (IRS = 6). Intriguingly, high expression of DDX19A was associated with lymph node metastasis and larger tumor size (Figure 1G, Supplementary Table 1). Furthermore, Kaplan–Meier survival plot revealed that patients with high DDX19A expression levels had an unfavorable prognosis relative to those with low DDX19A expression (Figure 1H). Moreover, TCGA data echoed the above survival correlation (Figure 1I). In summary, these data indicate that DDX19A may play a tumor-promoting role in CSCC.

### DDX19A Promotes the Metastasis of Cervical Squamous Cell Carcinoma Cells *in vitro* and *in vivo*

**Figure 2:**
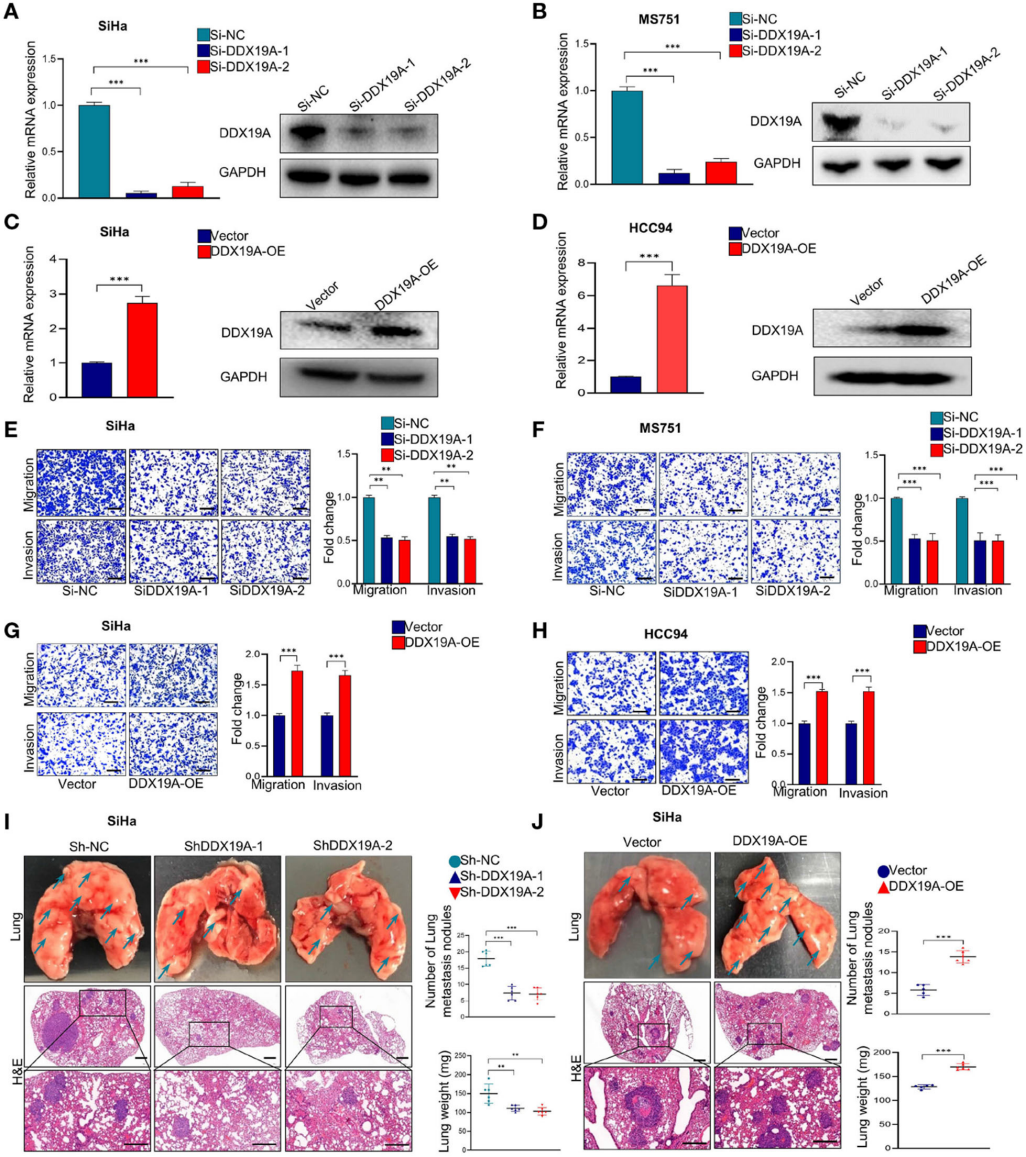
DEAD-box helicase 19A (DDX19A) promotes the metastasis of cervical squamous cell carcinoma (CSCC) cells *in vitro* and *in vivo*. **(A,B)** qRT-PCR and Western blot were employed to evaluate the efficacy of DDX19A mRNA and protein knockdown in SiHa and MS751 (*n* = 3). **(C,D)** qRT-PCR and Western blot were employed to evaluate the efficacy of DDX19A mRNA and protein overexpression in SiHa and HCC94 (*n* = 3). **(E,F)** Cell migration assay and Matrigel invasion assay were employed to investigate the effect of DDX19A knockdown in SiHa and MS751 cell migration and invasion ability (scale bar: 200 μm) (*n* = 3). **(G,H)** Cell migration assay and Matrigel invasion assay were employed to investigate the effect of DDX19A overexpression in SiHa and HCC94 cell migration and invasion ability (scale bar: 200 μm) (*n* = 3). **(I,J)** Arrows showed the representative results of metastatic lung nodules. H&E staining was used to stain metastatic lung nodules (200× and 400× magnification; scale bar: 200 μm). Dot plots showed the results of the number of lung metastasis nodules and lung weight (*n* = 6). Results represent three independent experiments **(A–H)**. The results were shown as means ± SD, **p < 0.01, ***p < 0.001 by two-tailed Student's *t*-test.

Using bioinformatics analysis and confirmatory experiments in clinical specimens, we found that the expression of DDX19A was positively correlated with tumor metastasis. Moreover, the occurrence of metastasis is an important reason for the poor prognosis of CSCC patients. To validate the function of DDX19A in CSCC metastasis, we conducted functional studies both *in vitro* and *in vivo*. Firstly, DDX19A expression was identified in various CSCC lines by Western blot (Supplementary Figure 1A). Subsequently, we knocked down DDX19A in two CSCC cell lines, SiHa (Figure 2A) and MS751 (Figure 2B), and overexpressed DDX19A in two cell lines, SiHa (Figure 2C) and HCC94 (Figure 2D). Transwell migration and cell invasion assays showed that knockdown of DDX19A suppressed the migration and invasion abilities of CSCC cells (Figures 2E,F), whereas, DDX19A upregulation markedly enhanced these functions (Figures 2G,H).

We further investigated whether DDX19A could affect tumor metastasis *in vivo*. Stable SiHa cell lines with DDX19A knockdown or overexpression were established and verified (Supplementary Figures 1B,C). The pulmonary metastasis nude mouse model was established by caudal intravenous injection of the above cell lines into nude mice. As shown in Figure 2I, compared with the control groups, DDX19A knockdown cells exhibited fewer lung metastases in nude mice, as measured by both the number of nodules and tumor weight. In contrast, overexpression of DDX19A significantly promoted lung metastasis (Figure 2J). Collectively, these results indicate that DDX19A may promote the metastasis of CSCC cells both *in vitro* and *in vivo*.

In addition, epithelial–mesenchymal transition (EMT) has been widely recognized as a critical mechanism of cancer metastasis (21–23). Thus, we further explored whether DDX19A affected EMT of CSCC cells. As shown in Supplementary Figure 1D, DDX19A knockdown in SiHa cells led to a significantly increased protein level of zonula occludens 1 (ZO-1) and E-cadherin and decreased expression of N-cadherin, Snail, and β-catenin. Therefore, these data indicate that DDX19A promotes the metastatic ability of CSCC cells by inducing EMT.

### DDX19A Regulates NOX1 Expression and Enhances Reactive Oxygen Species Production in Cervical Squamous Cell Carcinoma

**Figure 3:**
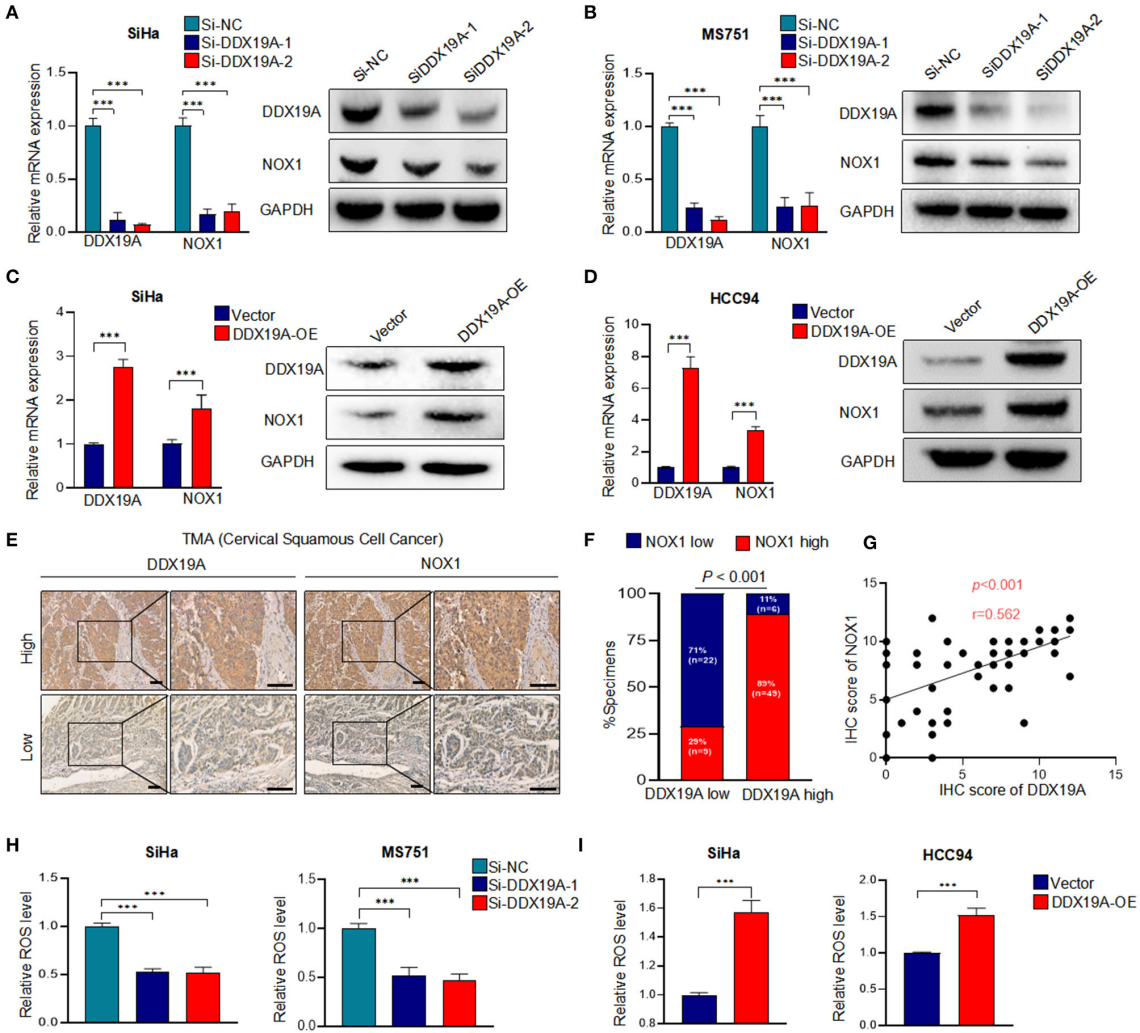
DEAD-box helicase 19A (DDX19A) regulates NADPH oxidase 1 (NOX1) expression and enhances reactive oxygen species (ROS) production in cervical squamous cell carcinoma (CSCC). **(A,B)** qRT-PCR and Western blot were performed to detect the mRNA and protein levels of NOX1 in DDX19A knockdown cells (SiHa and MS751) (*n* = 3). **(C,D)** qRT-PCR and Western blot were performed to detect the mRNA and protein levels of NOX1 in DDX19A-overexpressing cells (SiHa and HCC94) (*n* = 3). **(E)** Immunohistochemical (IHC) staining was performed to investigate the correlation between the protein levels of DDX19A and NOX1 in CSCC tissues (200× and 400× magnification; scale bar: 200 μm). **(F)** Correlation between DDX19A expression and NOX1 expression in patients. Chi-square test was used. **(G)** The correlation between the IHC scores of the DDX19A protein and the NOX1 protein was evaluated using Spearman rank analysis. **(H)** The 2′,7′-dichlorodihydrofluorescein diacetate (DCFH-DA) fluorescence assay was performed to investigate the effect of DDX19A knockdown on ROS production in SiHa and MS751 (*n* = 3). **(I)** The DCFH-DA fluorescence assay was performed to investigate the effect of DDX19A overexpression on ROS production in SiHa and HCC94 (*n* = 3). Results represent three independent experiments **(A–D,H,I)**. The results were shown as means ± SD, ***p < 0.001 by two-tailed Student's *t*-test.

It has been previously reported that DDX19A participates in the activation of the NOX1 promoter in TNF-α-induced A549 cells (19). NOX1, a member of the nicotinamide adenine dinucleotide phosphate (NADPH) oxidase family, is a key enzyme that regulates redox reactions in the body (24). It has been widely reported that the biological function of NOX1 is to produce ROS (25–27), and ROS is found to regulate the metastasis through extracellular signal-regulated kinase (ERK) signaling pathway and EMT in a variety of tumors (28–30). In cervical cancer, ROS can promote cervical cancer metastasis through the β-catenin–WNT signaling pathway (31) and EMT (32). We assumed that DDX19A promotes the expression of NOX1 increases the production of ROS. Therefore, we measured the expression of NOX1 in DDX19A-knockdown or -overexpressing cells. Both NOX1 mRNA and protein were decreased as the expression of DDX19A was inhibited in SiHa and MS751 (Figures 3A,B) and *vice versa* (Figures 3C,D).

Next, we examined the expression of NOX1 in CSCC tissues. As shown in Figures 3E,F, CSCC tissues with a higher DDX19A expression exhibited significantly increased staining intensity of NOX1 protein compared with those showing a lower DDX19A expression. A correlation analysis showed that the IHC score of DDX19A was positively correlated with that of NOX1 (Figure 3G). DDX19A knockdown resulted in reduced ROS production in SiHa and MS751 (Figure 3H), and DDX19A overexpression stimulated ROS production in SiHa and HCC94 (Figure 3I). Overall, these results confirmed that DDX19A regulated NOX1 expression and enhanced ROS production in CSCC.

### NOX1 Promotes Metastasis and Reactive Oxygen Species Production in Cervical Squamous Cell Carcinoma Cells and May Serve as a Prognostic Marker in Cervical Squamous Cell Carcinoma Patients

**Figure 4:**
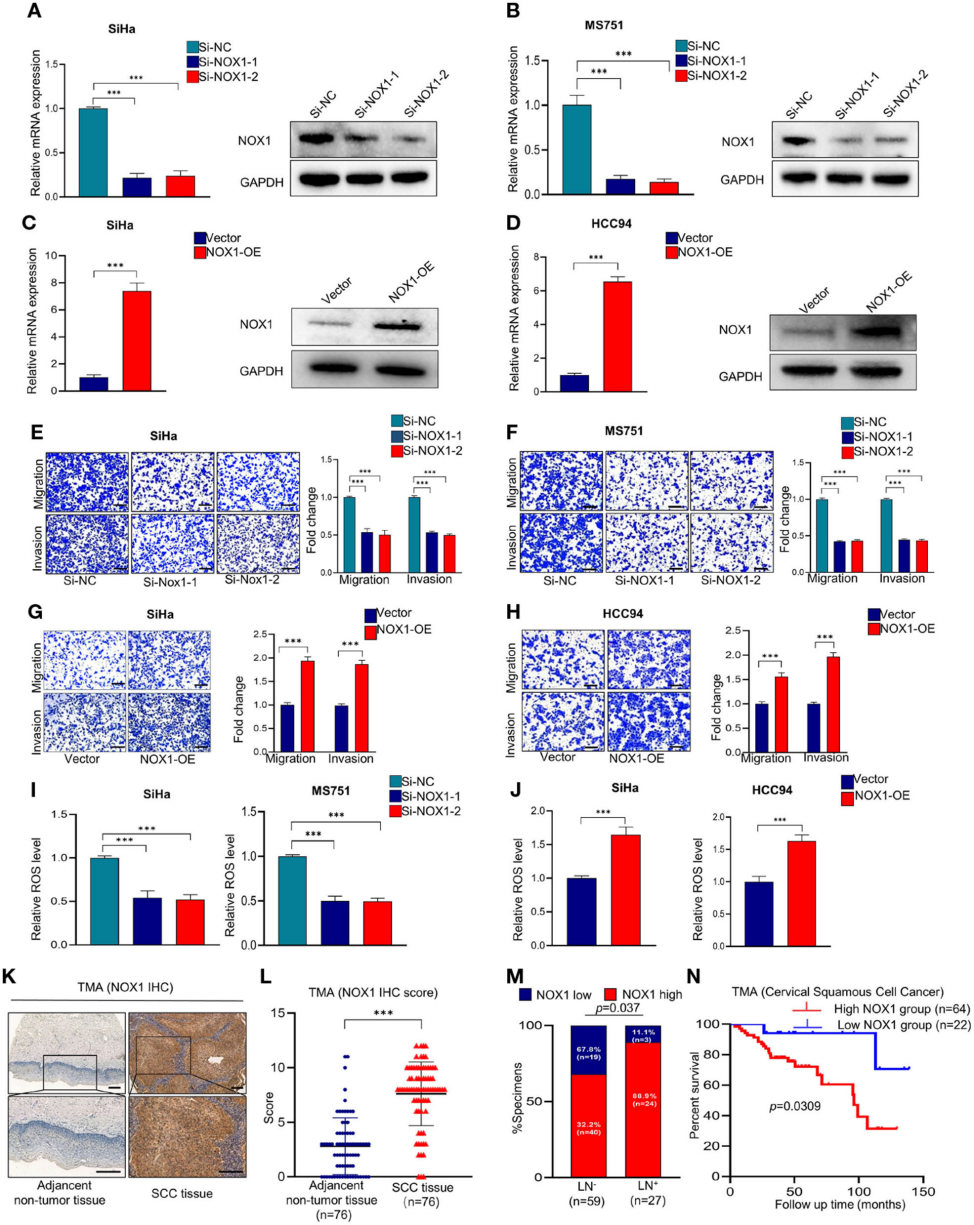
NADPH oxidase 1 (NOX1) promotes metastasis and reactive oxygen species (ROS) production in cervical squamous cell carcinoma (CSCC) cells and may serve as a prognostic marker in CSCC patients. **(A,B)** qRT-PCR and Western blot were employed to evaluate the knockdown efficacy of NOX1 in SiHa and MS751 (*n* = 3). **(C,D)** qRT-PCR and Western blot were employed to evaluate the overexpression efficacy of NOX1 in SiHa and HCC94 (*n* = 3). **(E,F)** Cell migration assay and Matrigel invasion assay were employed to investigate the effects of NOX1 knockdown cells (SiHa and MS751) (scale bar: 200 μm). **(G,H)** Cell migration assay and Matrigel invasion assay were employed to investigate the effects of NOX1-overexpressing cells (SiHa and HCC94) (scale bar: 200 μm). **(I)** ROS level was reduced in NOX1 knockdown cells (SiHa and MS751). **(J)** ROS level was increased in NOX1-overexpressing cells (SiHa and HCC94) (*n* = 3). **(K)** Representative images of the immunohistochemical (IHC) staining of NOX1 in human CSCC tissues and adjacent non-tumor tissues (scale bar: 200 μm). **(L)** Dot plots to show the IHC score of DDX19A expression using 76 pairs of CSCC tissues and adjacent non-tumor tissues tumor microarray (TMA) tissue sections (*p* < 0.001). **(M)** Correlation between lymph node metastasis and DDX19A expression in CSCC patients. Chi-square test was used. **(N)** Kaplan–Meier analysis was performed for our CSCC patients' cohort to evaluate the association between DDX19A protein level and 86 CSCC patients' overall survival. Results represent three independent experiments **(A–J)**. The results were shown as mean ± SD, ***p < 0.001 by two-tailed Student's *t*-test.

We further explored the effect of NOX1 on the function of CSCC. SiHa and MS751 were generated by transfection with NOX1 siRNA (Figures 4A,B). Inhibition of NOX1 expression arrested the migration and invasion of SiHa and MS751 cells (Figures 4E,F), whereas enhanced NOX1 expression (Figures 4C,D) promoted cell migration and invasion (Figures 4G,H). ROS production followed the same trend as NOX1 (Figures 4I,J). Taken together, these data demonstrated that NOX1 could promote cell migration and invasion and enhanced ROS production in CSCC cells.

To explore the clinical significance of NOX1, we performed IHC staining of NOX1 in CSCC TMA (Figure 4K) and demonstrated that NOX1 expression level was significantly elevated in CSCC tissues compared to adjacent non-tumor tissues (Figure 4L, Supplementary Table 3). Furthermore, NOX1 expression was associated with tumor size and lymph node metastasis (Figure 4M, Supplementary Table 4). Additionally, high expression of NOX1 significantly decreased overall survival (Figure 4N). Collectively, these data show that NOX1 may function as a prognostic marker for CSCC.

### The NOX1/Reactive Oxygen Species Axis Exerts a Pro-metastasis Effect Downstream of DDX19A

**Figure 5:**
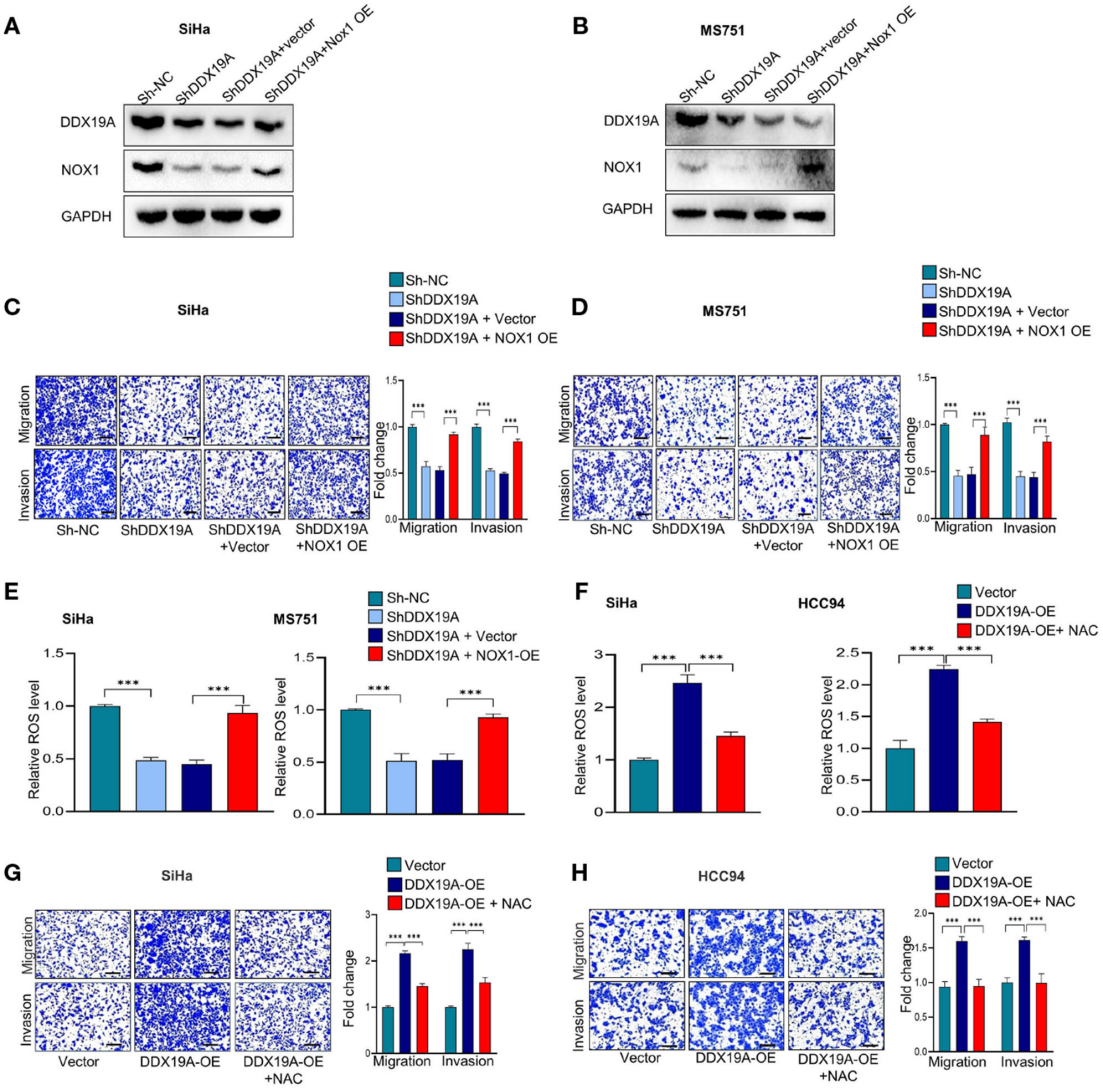
The NADPH oxidase 1 (NOX1)/reactive oxygen species (ROS) axis exerts a pro-metastasis effect downstream of DEAD-box helicase 19A (DDX19A). **(A,B)** Western blot was employed to evaluate the overexpression efficacy of NOX1 proteins in DDX19A knockdown cell lines (SiHa and MS751) (*n* = 3). **(C,D)** Cell migration assay and Matrigel invasion assay were performed to evaluate whether restoring NOX1 expression could increase cellular migration and invasion in DDX19A knockdown cell lines (SiHa and MS751) (*n* = 3). **(E)** The 2′,7′-dichlorodihydrofluorescein diacetate (DCFH-DA) fluorescence assay was performed to investigate whether NOX1 overexpression weakens the increase of ROS production induced by DDX19A knockdown in SiHa and MS751. **(F)** The DCFH-DA fluorescence assay was used to examine the ROS level in DDX19A-overexpressing cell lines (SiHa and HCC94) treated with or without N-acetylcysteine (NAC) (ROS inhibitor). **(G,H)** Cell migration assay and Matrigel invasion assay were performed to investigate whether NAC treatment could recover cell migration and invasion ability in DDX19A-overexpressing cell lines (SiHa and HCC94) (scale bar: 200 μm). Results represent three independent experiments. The results were shown as mean ± SD, ***p < 0.001 by two-tailed Student's *t*-test.

To further investigate whether NOX1/ROS signaling acts downstream of DDX19A in regulating CSCC metastasis, we performed a series of rescue experiments. Western blot analysis was used to evaluate the efficacy of NOX1 overexpression in DDX19A-knockdown SiHa and MS751 (Figures 5A,B). Overexpression of NOX1 reversed the reduction of cell migration and invasion (Figures 5C,D) as well as ROS production induced by DDX19A knockdown (Figure 5E). In addition, we treated HCC94 and SiHa cells with the ROS inhibitor, N-acetylcysteine (NAC), which effectively abolished ROS production induced by DDX19A overexpression (Figure 5F). Consequently, NAC treatment reversed the decrease in migration and invasion induced by DDX19A overexpression in HCC94 and SiHa cells (Figures 5G,H). In addition, treating SiHa cells with the ROS inhibitor NAC led to a significantly increased protein level of ZO-1 and E-cadherin and decreased the expression of N-cadherin, Snail, and β-catenin (Supplementary Figures 2A,B), while elevating ROS in DDX19A-knockdown SiHa can decrease the protein level of ZO-1 and E-cadherin and increase the expression of N-cadherin, Snail, and β-catenin (Supplementary Figures 2C,D). These data suggest that the NOX1/ROS axis exerts a metastasis-promoting effect downstream of DDX19A.

## Discussion

Tumor metastasis is considered the major cause for treatment failure and death in CSCC. However, the mechanisms underlying CSCC metastasis have not been fully understood. In this study, we identified DDX19A as a novel regulator of CSCC metastasis, which exerts pro-metastasis function *via* the NOX1/ROS pathway.

DDX19A was identified as a novel cytosolic RNA sensor that bridged porcine reproductive and respiration syndrome virus RNA and NLRP3 to activate the NLRP3 inflammasome (18). Moreover, a meta-analysis showed that a downsized four-gene signature, consisting of DDX19A, FOXM1, KPNA4, and H2AFV, represents a highly significant finding for the biology underlying histological grades in breast cancer, in particular, regarding cell proliferation, and DNA stability (33). However, the expression and function of DDX19A in cancer have not been reported. In this study, data from public databases and the results obtained from our clinical specimens consistently showed increased expression of DDX19A in CSCC, suggesting a tumor-promoting role in CSCC. Functional assays further confirmed that DDX19A exerted tumor-promoting roles in CSCC by stimulating cell metastasis.

A recent study has revealed that DDX19A participates in the activation of the NOX1 promoter in TNF-α-induced A549 cells (19). NOX1, a member of the NADPH oxidase family, is the main source of ROS production (34). Accumulating evidence suggests that NOX1 is involved in the occurrence and development of a variety of cancers (35). For example, NOX1 promoted cell metastasis by mediating ROS production in hepatocellular carcinoma (36). NOX1 regulated colorectal cancer metastasis by modulating the stability of a disintegrin and metalloprotease domain 17 (37). NOX1 could stimulate gastric carcinogenesis by enhancing inflammation or oxygen radical activity (38). However, the expression and function of NOX1 in CSCC remain undefined. We assume that DDX19A promotes the expression of NOX1 and thus enhances the production of ROS. Our results showed that DDX19A affected NOX1 expression in CSCC cell lines and that DDX19A expression was found positively correlated with NOX1 expression in CSCC specimens. DDX19A could promote migration and invasion of CSCC cells using both gain- and loss-of-function assays. Furthermore, overexpression of NOX1 reversed the influence of DDX19A downregulation on metastasis *in vivo*. Taken together, our data demonstrated that DDX19A could accelerate migration and invasion of CSCC cells *in vitro* and promote cancer cell metastasis *in vivo* via enhancing NOX1 expression. However, the underlying molecular mechanism of interaction between DDX19A and NOX1 in CSCC remains unknown, which is worthy of a future study.

As a product of NOX1, ROS levels were essential regulators in cancer growth, metastasis, and other malignant behaviors (39). Numerous researches have shown that ROS can affect the invasion and metastasis of different tumors through different ways. For example, in colon cancer, chloride intracellular channel 1 can regulate cancer cell migration and invasion through ROS–ERK–matrix metalloproteinase 2 (MMP2) pathway (40). In breast cancer, benzo[a]pyrene exposure leads to cancer cell migration and invasion through ROS–ERK–MMP9 axis signaling (30). In addition, ROS can influence the invasion, and migration of tumor cells in lung cancer (41), gastric cancer (28), and liver cancer (29) through the EMT process. In cervical cancer, ROS has been confirmed to regulate cervical cancer cell invasion and metastasis through induction of EMT, activation of Nrf2, and Wnt/β-catenin signaling (42–44). In this study, a novel regulator of ROS production, DDX19A, was identified. DDX19A knockdown reduced ROS production in SiHa and MS751 cells, while DDX19A overexpression resulted in the opposite effects in HCC94 and SiHa cells. Moreover, NAC treatment blocked the promoting effects of DDX19A on cell migration and invasion. Therefore, the regulatory effect of DDX19A on ROS production is responsible for the capabilities of cellular migration and invasion in CSCC cells with DDX19A overexpression.

In summary, the current study demonstrated that DDX19A contributed to metastasis of CSCC by inducing NOX1-mediated ROS production. Moreover, DDX19A/NOX1 may represent biomarkers of metastasis and novel therapeutic targets in CSCC patients.

## Data Availability Statement

The datasets presented in this study can be found in online repositories. The names of the repository/repositories and accession number(s) can be found in the article/Supplementary Material.

## Ethics Statement

The studies involving human participants were reviewed and approved by the Institutional Review Board of the Fifth Affiliated Hospital of Sun Yat-sen University (2020K78-1). The patients/participants provided their written informed consent to participate in this study. The animal study was reviewed and approved by Animal Experimentation Ethics Committee of the Fifth Affiliated Hospital of Sun Yat-sen University (Permission Number: 00083).

## Author Contributions

YJ, YL, BW, SC, HY, and JS performed the experiments. YW and FZ analyzed the data. CL and FW contributed reagents/materials/analysis tools. YJ wrote the paper. JL, YC, and HH conceived and designed the experiments. All authors read and approved the final manuscript.

## Conflict of Interest

The authors declare that the research was conducted in the absence of any commercial or financial relationships that could be construed as a potential conflict of interest.

## Supplementary Material

Supplementary Figure 1 DDX19A protein expression in human CSCC cell lines. **(A)** DDX19A protein expression in human cervical cancer cell lines was detected using Western blotting (*n* = 3). **(B,C)** Western blot was employed to evaluate the efficacy of DDX19A knockdown and overexpression in SiHa (*n* = 3). **(D)** Western blot were employed to evaluate the effect of DDX19A knockdown on the expression level of E-cadherin, N-cadherin, Snail, and β-catenin (*n* = 3).

Supplementary Figure 2 **(A)** DCFH-DA fluorescence assay was used to examine ROS level in SiHa treated with NAC (ROS inhibitor) (*n* = 3). **(B)** Western blot were employed to evaluate the expression level of E-cadherin, N-cadherin, Snail, and β-catenin in SiHa treated with NAC (ROS inhibitor) (*n* = 3). **(C)** DCFH-DA fluorescence assay was used to examine ROS level in DDX19A knockdown cells (SiHa) treated with H2O2 (*n* = 3). **(D)** Western blot were employed to evaluate the expression level of E-cadherin, N-cadherin, Snail, and β-catenin treated with H2O2 in DDX19A knockdown cells (SiHa) (*n* = 3).

Supplementary Table 1 Correlation analysis between the clinical features and DDX19A expression in CSCC.

Supplementary Table 2 DDX19A expression levels in different tissue specimens.

Supplementary Table 3 NOX1 expression levels in different tissue specimens.

Supplementary Table 4 Correlation analysis between the clinical features and NOX1 expression in CSCC.

## Abbreviations

TCGA: The Cancer Genome Atlas
GEO: Gene Expression Omnibus
CSCC: cervical squamous cell carcinoma
ATCC: American Type Culture Collection
NAC: N-acetylcysteine
DDX19A: DEAD-box helicase 19A
NOX1: NADPH oxidase 1
ROS: reactive oxygen species.
