# Corrigendum: DDX19A Promotes Metastasis of Cervical Squamous Cell Carcinoma by Inducing NOX1-Mediated ROS Production

**DOI:** 10.3389/fonc.2022.914765

**Published:** 2022-06-09

**Authors:** Yanhui Jiang, Baibin Wang, Yongliang Li, Jiahui Shen, Yutao Wei, Hanjie Li, Shangqiu Chen, Hua Yang, Famin Zeng, Changqing Liu, Feng Wang, Huanhuan He, Yong Chen, Jihong Liu

**Affiliations:** ^1^ Department of Gynecology, The Fifth Affiliated Hospital of Sun Yat-sen University, Zhuhai, China; ^2^ Guangdong Provincial Key Laboratory of Biomedical Imaging and Guangdong Provincial Engineering Research Center of Molecular Imaging, The Fifth Affiliated Hospital of Sun Yat-sen University, Zhuhai, China; ^3^ Department of Pathology, The Fifth Affiliated Hospital of Sun Yat-sen University, Zhuhai, China; ^4^ Department of Interventional Medicine, The Fifth Affiliated Hospital of Sun Yat-sen University, Zhuhai, China; ^5^ Department of Gynecologic Oncology, State Key Laboratory of Oncology in South China, Sun Yat-sen University Cancer Center, Guangzhou, China

**Keywords:** DDX19A, cervical squamous cell carcinoma, metastasis, Nox1, reactive oxygen species

In the original article, there were errors in **Figures 2C, D**, **4C** and **5D** as published. Wrong Western blot pictures were accidentally used in the **Figures 2C, D** and **4C**. The corrected **Figure 2** and **Figure 4** appear below. There was also an error in the images used in **Figure 5D**. The corrected **Figure 5** appears below.

**Figure 2:**
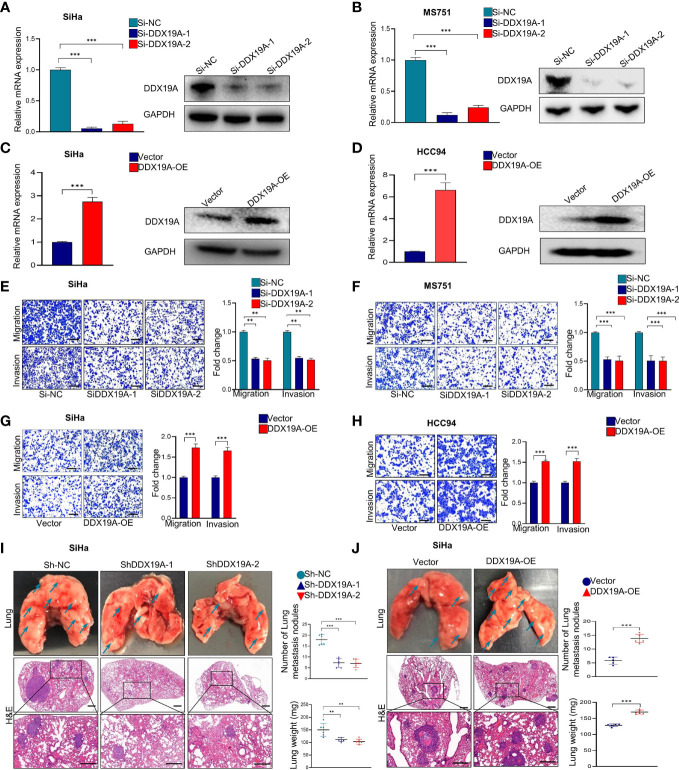
DEAD-box helicase 19A (DDX19A) promotes the metastasis of cervical squamous cell carcinoma (CSCC) cells *in vitro* and *in vivo*. **(A, B)** qRT-PCR and Western blot were employed to evaluate the efficacy of DDX19A mRNA and protein knockdown in SiHa and MS751 (n = 3). **(C, D)** qRT-PCR and Western blot were employed to evaluate the efficacy of DDX19A mRNA and protein overexpression in SiHa and HCC94 (n = 3). **(E, F)** Cell migration assay and Matrigel invasion assay were employed to investigate the effect of DDX19A knockdown in SiHa and MS751 cell migration and invasion ability (scale bar: 200μm) (n = 3). **(G, H)** Cell migration assay and Matrigel invasion assay were employed to investigate the effect of DDX19A overexpression in SiHa and HCC94 cell migration and invasion ability (scale bar: 200μm) (n = 3). **(I, J)** Arrows showed the representative results of metastatic lung nodules. H&E staining was used to stain metastatic lung nodules (200× and 400× magnification; scale bar: 200μm). Dot plots showed the results of the number of lung metastasis nodules and lung weight (n = 6). Results represent three independent experiments **(A–H)**. The results were shown as means ± SD, **p < 0.01, ***p < 0.001 by two-tailed Student’s t-test.

**Figure 4:**
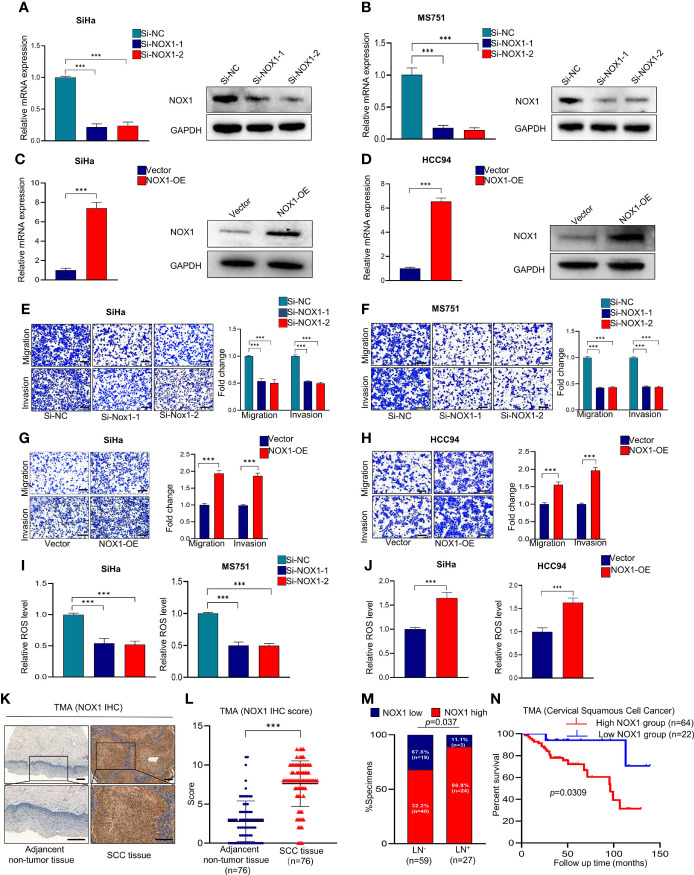
NADPH oxidase 1 (NOX1) promotes metastasis and reactive oxygen species (ROS) production in cervical squamous cell carcinoma (CSCC) cells and may serve as a prognostic marker in CSCC patients. **(A, B)** qRT-PCR and Western blot were employed to evaluate the knockdown efficacy of NOX1 in SiHa and MS751 (n = 3). **(C, D)** qRT-PCR and Western blot were employed to evaluate the overexpression efficacy of NOX1 in SiHa and HCC94 (n = 3). **(E, F)** Cell migration assay and Matrigel invasion assay were employed to investigate the effects of NOX1 knockdown cells (SiHa and MS751) (scale bar: 200μm). **(G, H)** Cell migration assay and Matrigel invasion assay were employed to investigate the effects of NOX1-overexpressing cells (SiHa and HCC94) (scale bar: 200μm). **(I)** ROS level was reduced in NOX1 knockdown cells (SiHa and MS751). **(J)** ROS level was increased in NOX1-overexpressing cells (SiHa and HCC94) (n =3). **(K)** Representative images of the immunohistochemical (IHC) staining of NOX1 in human CSCC tissues and adjacent non-tumor tissues (scale bar: 200μm). **(L)** Dot plots to show the IHC score of DDX19A expression using 76 pairs of CSCC tissues and adjacent non-tumor tissues tumor microarray (TMA) tissue sections (*p* < 0.001). **(M)** Correlation between lymph node metastasis and DDX19A expression in CSCC patients. Chi-square test was used. **(N)** Kaplan–Meier analysis was performed for our CSCC patients’ cohort to evaluate the association between DDX19A protein level and 86 CSCC patients’ overall survival. Results represent three independent experiments **(A–J)**. The results were shown as mean ± SD, ***p < 0.001 by two-tailed Student’s t-test.

**Figure 5:**
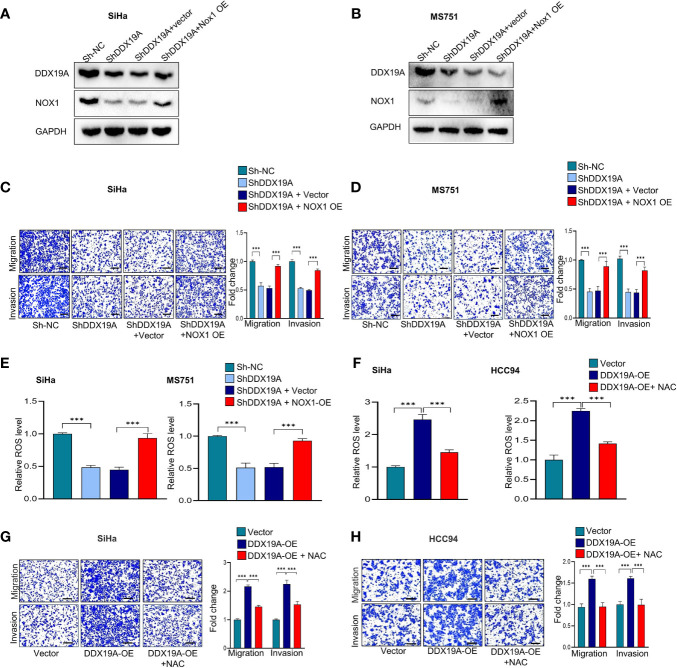
The NADPH oxidase 1 (NOX1)/reactive oxygen species (ROS) axis exerts a pro-metastasis effect downstream of DEAD-box helicase 19A (DDX19A). **(A, B)** Western blot was employed to evaluate the overexpression efficacy of NOX1 proteins in DDX19A knockdown cell lines (SiHa and MS751) (n = 3). **(C, D)** Cell migration assay and Matrigel invasion assay were performed to evaluate whether restoring NOX1 expression could increase cellular migration and invasion in DDX19A knockdown cell lines (SiHa and MS751) (n = 3). **(E)** The 2′,7′-dichlorodihydrofluorescein diacetate (DCFH-DA) fluorescence assay was performed to investigate whether NOX1 overexpression weakens the increase of ROS production induced by DDX19A knockdown in SiHa and MS751. **(F)** The DCFH-DA fluorescence assay was used to examine the ROS level in DDX19A-overexpressing cell lines (SiHa and HCC94) treated with or without N-acetylcysteine (NAC) (ROS inhibitor). **(G, H)** Cell migration assay and Matrigel invasion assay were performed to investigate whether NAC treatment could recover cell migration and invasion ability in DDX19A-overexpressing cell lines (SiHa and HCC94) (scale bar: 200μm). Results represent three independent experiments. The results were shown as mean ± SD, ***p < 0.001 by two-tailed Student’s t-test.

The authors apologize for these errors and state that this does not change the scientific conclusions of the article in any way. The original article has been updated.

## Publisher’s Note

All claims expressed in this article are solely those of the authors and do not necessarily represent those of their affiliated organizations, or those of the publisher, the editors and the reviewers. Any product that may be evaluated in this article, or claim that may be made by its manufacturer, is not guaranteed or endorsed by the publisher.

