# Supplementary figures and images for: DDX19A Promotes Metastasis of Cervical Squamous Cell Carcinoma by Inducing NOX1-Mediated ROS Production
Source: Front Oncol. 2021 Apr 22;11:629974. doi: 10.3389/fonc.2021.629974 (PMC8100682; doi:10.3389/fonc.2021.629974)

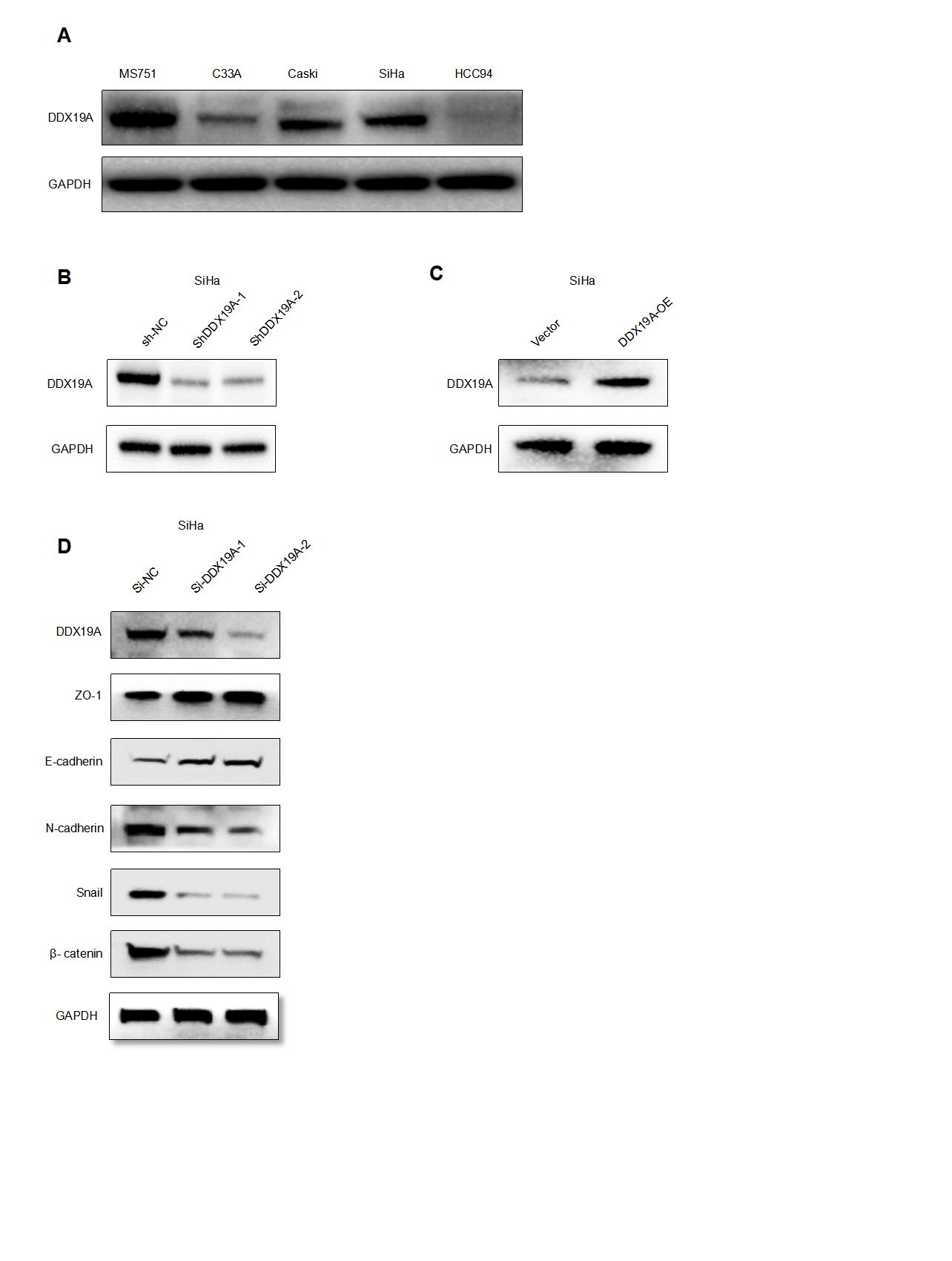

Supplement: Supplementary Figure 1 — DDX19A protein expression in human CSCC cell lines. (A) DDX19A protein expression in human cervical cancer cell lines was detected using Western blotting (n = 3). (B,C) Western blot was employed to evaluate the efficacy of DDX19A knockdown and overexpression in SiHa (n = 3). (D) Western blot were employed to evaluate the effect of DDX19A knockdown on the expression level of E-cadherin, N-cadherin, Snail, and β-catenin (n = 3). [file Image_1.TIF]

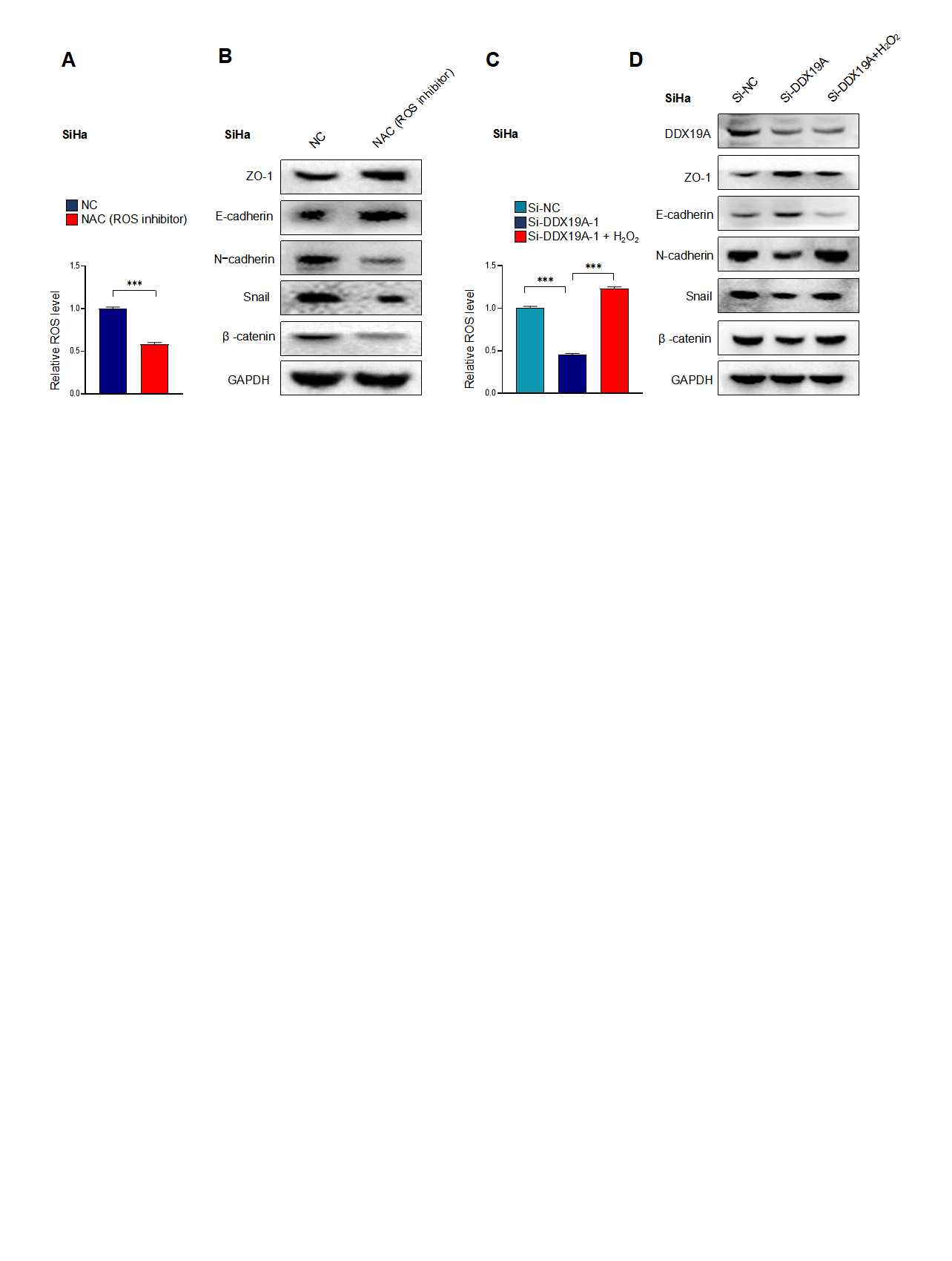

Supplement: Supplementary Figure 2 — (A) DCFH-DA fluorescence assay was used to examine ROS level in SiHa treated with NAC (ROS inhibitor) (n = 3). (B) Western blot were employed to evaluate the expression level of E-cadherin, N-cadherin, Snail, and β-catenin in SiHa treated with NAC (ROS inhibitor) (n = 3). (C) DCFH-DA fluorescence assay was used to examine ROS level in DDX19A knockdown cells (SiHa) treated with H2O2 (n = 3). (D) Western blot were employed to evaluate the expression level of E-cadherin, N-cadherin, Snail, and β-catenin treated with H2O2 in DDX19A knockdown cells (SiHa) (n = 3). [file Image_2.TIF]
