# Supplementary material for: DDX19A Promotes Metastasis of Cervical Squamous Cell Carcinoma by Inducing NOX1-Mediated ROS Production
Source: Front Oncol. 2021 Apr 22;11:629974. doi: 10.3389/fonc.2021.629974 (PMC8100682; doi:10.3389/fonc.2021.629974)
Supplement: Supplementary Table 1 — Correlation analysis between the clinical features and DDX19A expression in CSCC. [file Table_1.DOCX]

**Supplementary Table S1：**Correlation analysis between the clinical features and DDX19A expression in CSCC

| **Characteristics** | **Total** | **(%)** | **DDX19A** | | **p-value** |
| --- | --- | --- | --- | --- | --- |
|  |  |  | **Low(n=31 )** | **High(n= 55)** |  |
| **Age(years)** | 86 |  |  |  | 0.67 |
| **≤40** | 26 | （30.23） | 12 | 14 |  |
| **>40** | 60 | （69.77） | 29 | 41 |  |
| **FIGO stage** |  |  |  |  | 0.268 |
| **I** | 50 | （58.14） | 20 | 29 |  |
| **II** | 32 | （41.86） | 10 | 22 |  |
| **III** | 4 |  | 0 | 4 |  |
| **Tumor size (cm)** |  |  |  |  | 0.006 |
| **≤4** | 56 | （65.12） | 26 | 30 |  |
| **>4** | 30 | （34.89） | 5 | 25 |  |
| **Parametrial infiltration** |  |  |  |  | 0.361 |
| **Yes** | 9 | （10.47） | 2 | 7 |  |
| **No** | 77 | （89.53） | 29 | 48 |  |
| **Lymphovascular space invasion** |  |  |  |  | 0.616 |
| **Yes** | 25 | （22.09） | 8 | 17 |  |
| **No** | 61 | （77.91） | 23 | 38 |  |
| **Lymph node metastasis** |  |  |  |  | 0.011 |
| **Yes** | 27 | （31.40） | 5 | 22 |  |
| **No** | 59 | （68.60） | 26 | 33 |  |
