# Supplementary material for: DDX19A Promotes Metastasis of Cervical Squamous Cell Carcinoma by Inducing NOX1-Mediated ROS Production
Source: Front Oncol. 2021 Apr 22;11:629974. doi: 10.3389/fonc.2021.629974 (PMC8100682; doi:10.3389/fonc.2021.629974)
Supplement: Supplementary Table 2 — DDX19A expression levels in different tissue specimens. [file Table_2.DOCX]

**Supplementary Table S2: DDX19A expression levels in different tissue specimen**

| **Specimens** | **Total** | **DDX19A Staining** | | ***P*** |
| --- | --- | --- | --- | --- |
|  |  | **Negative No. (%)** | **Positive, No. (%)** |  |
| Normal | 76 | 62(81.58) | 14(18.42) |  |
| Carcinoma | 86 | 31(36.05) | 55(63.95) | <0.001 |

Pearson 2-tailed chi-square test was used to determine the statistical significance of the level of expression of DDX19A in different tissue specimens.
