# Supplementary material for: DDX19A Promotes Metastasis of Cervical Squamous Cell Carcinoma by Inducing NOX1-Mediated ROS Production
Source: Front Oncol. 2021 Apr 22;11:629974. doi: 10.3389/fonc.2021.629974 (PMC8100682; doi:10.3389/fonc.2021.629974)
Supplement: Supplementary Table 3 — NOX1 expression levels in different tissue specimens. [file Table_3.DOCX]

**Supplementary Table S3: NOX1 expression levels in different tissue specimen**

| **Specimens** | **Total** | **NOX1 Staining** | | ***P*** |
| --- | --- | --- | --- | --- |
|  |  | **Negative No. (%)** | **Positive, No. (%)** |  |
| Normal | 76 | 61(80.26) | 15(19.74) |  |
| Carcinoma | 86 | 17(19.76) | 69(80.23) | <0.001 |

Pearson 2-tailed chi-square test was used to determine the statistical significance of the level of expression of NOX1 in different tissue specimens.
