# Supplementary material for: DDX19A Promotes Metastasis of Cervical Squamous Cell Carcinoma by Inducing NOX1-Mediated ROS Production
Source: Front Oncol. 2021 Apr 22;11:629974. doi: 10.3389/fonc.2021.629974 (PMC8100682; doi:10.3389/fonc.2021.629974)
Supplement: Supplementary Table 4 — Correlation analysis between the clinical features and NOX1 expression in CSCC. [file Table_4.DOCX]

**Supplementary Table S4:** Correlation analysis between the clinical features and NOX1 expression in CSCC

| **Characteristics** | **Total** | **(%)** | **NOX1** | | **p-value** |
| --- | --- | --- | --- | --- | --- |
|  |  |  | **Low(n=22)** | **High(n=64)** |  |
| **Age(years)** | 86 |  |  |  | 0.000 |
| **≤40** | 26 | （30.23） | 18 | 8 |  |
| **>40** | 60 | （69.77） | 4 | 56 |  |
| **FIGO stage** |  |  |  |  | 0.096 |
| **I** | 50 | （58.14） | 6 | 44 |  |
| **II** | 32 | （41.86） | 14 | 18 |  |
| **III** | 4 |  | 2 | 2 |  |
| **Tumor size (cm)** |  |  |  |  | 0.015 |
| **≤4** | 56 | （65.12） | 19 | 37 |  |
| **>4** | 30 | （34.89） | 3 | 27 |  |
| **Parametrial infiltration** |  |  |  |  | 0.525 |
| **Yes** | 9 | （10.47） | 3 | 6 |  |
| **No** | 77 | （89.53） | 18 | 58 |  |
| **Lymphovascular space invasion** |  |  |  |  | 0.741 |
| **Yes** | 25 | （22.09） | 7 | 18 |  |
| **No** | 61 | （77.91） | 15 | 46 |  |
| **Lymph node metastasis** |  |  |  |  | 0.037 |
| **Yes** | 27 | （31.40） | 3 | 24 |  |
| **No** | 59 | （68.60） | 19 | 40 |  |
